# New Advanced Imaging Parameters and Biomarkers—A Step Forward in the Diagnosis and Prognosis of TTR Cardiomyopathy

**DOI:** 10.3390/jcm11092360

**Published:** 2022-04-22

**Authors:** Roxana Cristina Rimbas, Anca Balinisteanu, Stefania Lucia Magda, Simona Ionela Visoiu, Andrea Olivia Ciobanu, Elena Beganu, Alina Ioana Nicula, Dragos Vinereanu

**Affiliations:** 1 Cardiology and Cardiovascular Surgery Department, University and Emergency Hospital, 050098 Bucharest, Romania; roxanasisu@gmail.com (R.C.R.); anca.balinisteanu@ymail.com (A.B.); andreaciobanu7@yahoo.com (A.O.C.); beganu.elena@yahoo.com (E.B.); vinereanu@gmail.com (D.V.); 2 Cardiology Department, University of Medicine and Pharmacy Carol Davila, 020021 Bucharest, Romania; calin_simona_ionela@yahoo.com (S.I.V.); alinapavel74@yahoo.com (A.I.N.); 3 Radiology Department, University and Emergency Hospital, 050098 Bucharest, Romania

**Keywords:** TTR amyloidosis, new biomarkers, speckle-tracking echocardiography, cardiac scintigraphy, cardiac magnetic resonance, SPECT, PET, prognosis

## Abstract

Transthyretin amyloid cardiomyopathy (ATTR-CM) is an infiltrative disorder characterized by extracellular myocardial deposits of amyloid fibrils, with poor outcome, leading to heart failure and death, with significant treatment expenditure. In the era of a novel therapeutic arsenal of disease-modifying agents that target a myriad of pathophysiological mechanisms, timely and accurate diagnosis of ATTR-CM is crucial. Recent advances in therapeutic strategies shown to be most beneficial in the early stages of the disease have determined a paradigm shift in the screening, diagnostic algorithm, and risk classification of patients with ATTR-CM. The aim of this review is to explore the utility of novel specific non-invasive imaging parameters and biomarkers from screening to diagnosis, prognosis, risk stratification, and monitoring of the response to therapy. We will summarize the knowledge of the most recent advances in diagnostic, prognostic, and treatment tailoring parameters for early recognition, prediction of outcome, and better selection of therapeutic candidates in ATTR-CM. Moreover, we will provide input from different potential pathways involved in the pathophysiology of ATTR-CM, on top of the amyloid deposition, such as inflammation, endothelial dysfunction, reduced nitric oxide bioavailability, oxidative stress, and myocardial fibrosis, and their diagnostic, prognostic, and therapeutic implications.

## 1. Introduction

Transthyretin cardiomyopathy (ATTR-CM) is an infiltrative process of extracellular myocardial deposits of amyloid fibrils, generating heart failure (HF) and death [1,2,3]. Some modifying agents are now available, but their usefulness in the amyloidosis treatment is dependent on the stage of the disease. In this light, ATTR-CM diagnosis is very important to be done in the early phases. Moreover, it is crucial to define new markers, which will allow us to select better patients for different types of treatment [1,3].

ATTR-CM has two distinct subtypes: familial TTR (mutant) (ATTRm) and wild-type TTR (ATTRwt) [4]. ATTR is derived from TTR, a carrier of thyroxine and retinol bound to retinol-binding protein (RBP), which is produced mainly by the liver. Amyloid generation is stimulated by either one of the 120 known point mutations in its gene located on the long arm of chromosome 18, or by unknown mechanisms related to aging in ATTRwt [5]. Multiple organs may be affected, but cardiac involvement is the main predictor of survival [3]. ATTR-CM results in a step-by-step hypertrophic remodeling and finally restrictive cardiomyopathy. Diagnosis of ATTR-CM is challenging and requires a high degree of clinical suspicion and integration of cardiac imaging, biomarkers, histologic and molecular markers [6]. The optimal diagnosis strategy requires a multidisciplinary approach, [7,8].

The ‘gold standard’ diagnostic method is a histopathological one. Amyloid fibril type is confirmed by mass spectroscopy or immunohistochemistry of biopsy material. However, non-invasive imaging techniques add important data, supporting the new markers derived from cardiac scintigraphy, speckle-tracking-echocardiography (2DSTE), cardiac magnetic resonance (CMR), and the diagnosis of ATTR-CM [7,8,9]. New imaging protocols are in continuous change to achieve a better quantification of the CA and a prediction of prognosis. Moreover, serum cardiac biomarkers such as Troponin (Tp) and BNP/NTproBNP, are frequently used for risk stratification [7,8,9,10].

New potential mechanisms involved in the pathophysiology of ATTR-CM have been proposed. Recent data suggest that inflammation, oxidative stress, reduced NO bioavailability, endothelial dysfunction, myocardial remodeling, and fibrosis might play an important role in ATTR pathogenesis, on top of amyloid infiltration [3,11,12]. Subsequently, the use of new biomarkers might provide input from different pathophysiological pathways that could add important data in the diagnosis and also in the assessment of the prognosis of ATTR-CM, identifying patients who are at high risk of ATTR-CM progression [3,9].

Although important developments in the field of ATTR-CM were achieved, there are still important unanswered questions: (1) the optimal imaging and biomarker protocols for screening of asymptomatic variant TTR carriers; (2) the understanding of unclear diagnostic features in cardiac amyloidosis (CA), and (3) the best markers (imaging and/or biological) for monitoring response to therapy in ATTR-CM [9].

Accordingly, our review will summarize the most recent advances in imaging protocols and new biomarkers, integrated at all levels of the clinical algorithm of ATTR-CM, from screening to diagnosis, prognosis, and treatment tailoring.

## 2. Clinical Red Flags in ATTR-CM 

CA has a high mortality rate and high clinical heterogeneity, resulting in a delayed diagnosis. Active screening for CA diagnosis becomes mandatory, as a potentially treatable disease, using new biomarkers and imaging modalities. This might be a better strategy because early identification of ATTR-CM can maximize the benefit of therapy, improving outcomes [3,13,14,15]. 

In patients with HFpEF, the prevalence was reported from 13% to 17% [6]. In patients with aortic stenosis (AS) undergoing transcatheter aortic valve implantation (TAVI), approximately 14–16 % have occult CA [15,16,17]. Moreover, the prevalence of ATTRwt is estimated to be 10–25% in people over the age of 80 [18,19].

A list of “**red flags**” has been proposed by experts to raise the suspicion of ATTR-CM and lead to further investigations [9,20,21]. 

**Cardiac manifestations**. ATTR-CM consists mainly of symptoms of HF generated by biventricular wall stiffness and restrictive cardiomyopathy, arrhythmia, or sudden death [9,22,23]. The diagnosis is frequently challenging when patients present only with nonspecific cardiac signs and symptoms, particularly in the absence of systemic manifestations, [23,24,25,26,27]. Some patients present with syncope or pre-syncope generated by low stroke volume and autonomic dysfunction, which is associated with poor prognosis [3,5,25,27,28]. Atrial involvement generates atrial thrombosis and systemic embolism, even in patients with sinus rhythm [9,24,25,29]. Typical angina may occur, without significant atherosclerotic epicardial coronary disease, being generated by the perivascular infiltration [29,30,31]. Patients are often diagnosed with HFpEF, which can appear phenotypically as hypertrophic cardiomyopathy [6,19,25,32].

**Extracardiac manifestations.** CA is typically preceded by extracardiac manifestations, which can represent significant clues for diagnosis. Ophthalmologic, orthopedic, neurologic, and gastrointestinal abnormalities may be warning signs [6,7,9,33,34]. Bilateral carpal tunnel syndrome is the most common noncardiac manifestation, and it can precede clinical HFpEF symptoms by 5 to 10 years [35]. Lumbar spinal stenosis may be associated mainly with ATTRwt [36]. Polyneuropathy consists of sensory abnormalities (paresthesia, numbness in the feet, pain, or a sensation of “walking on rolled-up socks”), autonomic dysfunction (digestive and erectile dysfunction) [22], and, in advanced disease, motor polyneuropathy with loss of reflexes [22,35,36]. Table 1 summarizes red flags suggestive of ATTR-CM, with systemic and cardiac infiltration.

**Figure 1:**
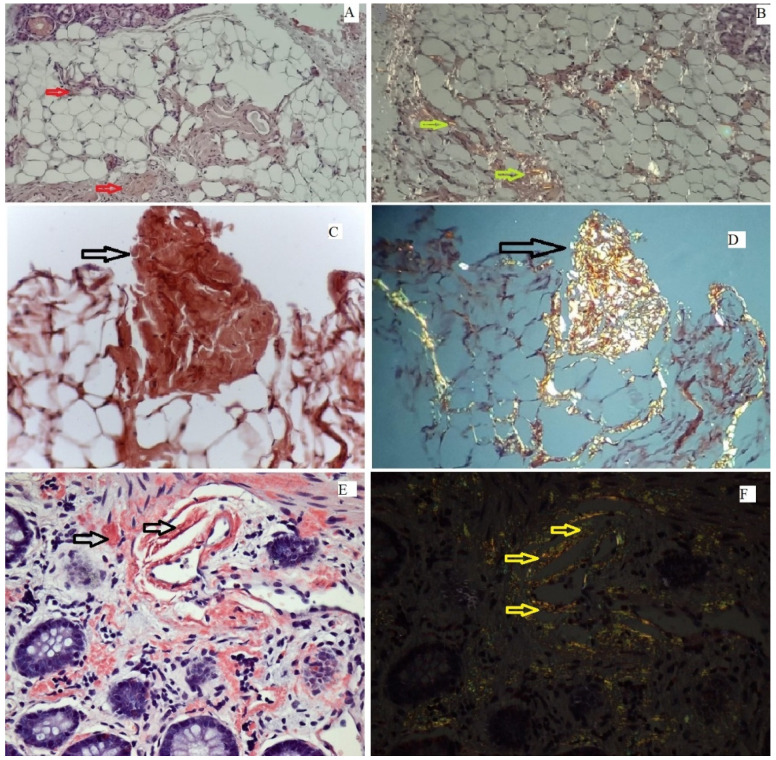
**Anatomopathological main findings suggestive of cardiac amyloidosis**. (**Upper panel**). Salivary glands biopsy, showing Congo red positivity (red arrows) (**A**), with apple green birefringence on polarization yellow arrows (**B**); (**Mid panel**). **Abdominal fat pad,** showing important Congo red positivity (black arrow) (**C**), with apple green birefringence on polarization (black arrow) (**D**); (**Lower panel**). **Rectal biopsy,** with important and diffuse infiltration with amyloid fibrils, Congo red positivity (black arrows) (**E**), with apple green birefringence on polarization (yellow arrows) (**F**).

One of the most critical steps in the evaluation protocol of the patient with suspected ATTR-CM is the identification of the amyloid type since a mistaken diagnosis can have disastrous consequences. Undetected or misdiagnosed ATTR-CM leads to disease progression and worse outcomes. The gold standard for the diagnosis of ATTR-CM is an endomyocardial biopsy (EMB). However, it may fail to diagnose amyloid infiltration. Since it is an invasive procedure, it can lead to serious complications: cardiac tamponade and even death [38,39]. Therefore, biopsies from other affected organs, such as salivary glands, abdominal fat, and duodenal or rectal ones, are more frequently used to confirm CA [9,19,20] (Figure 1).

## 3. Electrocardiography in ATTR-CM

Electrocardiography (ECG) is a feasible and cost-efficient tool to assess CA. ECG changes highly suggestive of CA are summarized in Table 1 [9,13,15,40]. The optimal follow-up plan in patients with CA is not established yet. Guidelines suggest a 6-month visit with ECG, NT-proBNP, and troponin), yearly echocardiography, and 24 h ambulatory ECG monitoring. ATTRm asymptomatic genetic carriers should have a yearly ECG and a biannual 24 h ambulatory ECG monitoring [7,9,11].

The prevalence of specific electrocardiographic findings has been well reviewed in patients with AL-CA but is still under research in the ATTR-CM [41]. The THAOS registry, the largest available database (425 patients) in ATTR-CM added some important information, including ECG patterns [42]. Low voltage on limb leads is the classic ECG finding in CA, followed by pseudo-infarct pattern and atrioventricular conduction disturbances [43,44,45].

The most frequently used definitions of ***low voltage*** are a QRS amplitude ≤ 0.5 mV in all limb leads, ≤ 1 mV in all precordial leads, or a Sokolow index ≤ 1.5 mV. Low voltage is present in the majority of AL cases, whereas only one-third of patients with CA have low voltage on the ECG [42,43]. Data from the THAOS registry suggested that the prevalence is significantly higher in moderate to severe LVH evaluated by 2DE, compared to mild LVH, and also in ATTR wt. 

***Pseudo-infarct patterns***, defined as QS waves in two concordant leads, are described in 47–60% of all CA patients and have the following distribution: anterior (36%), inferior (12%), and lateral (14%) [45,46]. Data from the THAOS registry suggested that the prevalence of Q waves was similar in the late Val30Met, non-Val30Met ATTRm, and ATTRwt groups, being significantly higher in moderate to severe LVH compared to mild LVH [42]. Several studies have reported the association between low voltage and pseudo-infarct patterns, with a prevalence of 25% [45,47]. However, the absence of both signs should not rule out the diagnosis of ATTR-CM [9].

***Arrhythmias*** occur often in patients with ATTR-CM, the most common being atrial fibrillation and flutter. ***Intraventricular conduction delays and blocks*** seem to be more frequent in ATTR-CM compared to the AL subtype. Intraventricular conduction blocks, and most importantly their progression over time, suggest disease progression and are associated with higher mortality [48,49]. ***Fragmented QRS***, defined as various RSR′ patterns, reflect regional myocardial scars and worse prognosis [50]. ***Decreased heart rate variability (HRV)*** was reported in ATTRm, associated with autonomic dysfunction. Reduced HRV on a 24 h Holter is associated with higher short-term mortality in the AL subtype [48,49]. A Sokolow–Lyon index <1.5 mm associated with QTc duration >440 msec was found to have a sensitivity of 85% and a specificity of 100% for the diagnosis of CA [51]. ***LVH***, although atypical, has been reported in some studies of biopsy-proven CA [45]. LVH might be present in CA, especially in the ATTR, and should not exclude the CA diagnosis [11].

The relation between ECG findings and outcomes is still under research. Low QRS voltage was associated with poor survival in both AL and ATTR-CM [46,52]. A Sokolow index ≤ 1.5 mV was predictive of a combined outcome of time to hospitalization, heart transplant, or death in both AL and ATTR-CM [41]. A multicenter retrospective study investigated the association between ECG parameters, myocardial deformation by STE, NT-proBNP, and prognosis in a cohort with AL-CA. ECG scores consisting of the presence of prolonged QTc (≥483 msec), and abnormal QRS axis showed a good association with longitudinal LV dysfunction and NT-proBNP, suggesting that ECG findings might provide prognostic additional information in CA [53]. Moreover, in patients with confirmed CA, both AL and ATTR, a higher LV mass–voltage ratio (calculated using CMR derived LV mass and ECG) seems to be related to HF hospitalization, even after adjusting for other clinical covariates such as NYHA class, BNP, LVEF and extracellular volume by CMR [54]. Unfortunately, data regarding the prognostic value of ECG findings in ATTR-CM, as well as the progression/regression of ECG in treated ATTR-CM patients, are lacking.

## 4. Echocardiography in ATTR Cardiac Amyloidosis

***Conventional 2D echocardiography.*** 2DTTE is a widely available, cost-efficient, and non-irradiating investigation, which describes cardiac structure and function, detecting increased LV wall thickness, the abnormal sparkling aspect of the myocardium, and provides details about diastolic and systolic function [8,9]. Unfortunately, most cardiac morphological changes are obvious only in more advanced stages of CA. 2DTTE adds important data in patients with HFpEF, restrictive or non-obstructive hypertrophic cardiomyopathies, and disproportionated hypertrophy. More than 90% of patients with CA have hypertrophy with a small LV cavity size. Currently used echocardiographic criteria are validated only for AL-CA and have reasonable sensitivity but low specificity [55].

**Figure 2:**
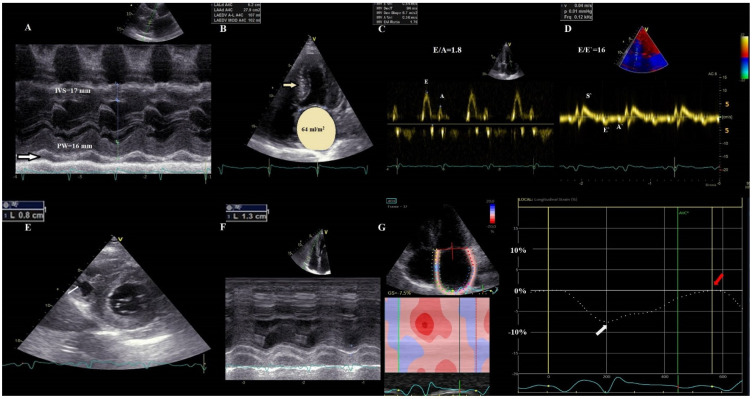
**Transthoracic echocardiographic images in advanced ATTR-CM**. (**A**) M−mode parasternal long-axis view of the LV displaying concentric severe LV hypertrophy (LV mass = 157 g/m^2^, RWT = 0.8), with mild pericardial effusion (white arrow); (**B**) apical 4-chamber view demonstrating a sparkling texture of the IVS and severely dilated LA (64 mL/m^2^); (**C**) Pseudo normal transmitral filling pattern (E/A = 1.8); (**D**) TDI tracing of the mitral annulus showing that all tissue velocities are <5 cm/s, with increased E/E’ ratio. (**E**) parasternal short axis view showing biventricular hypertrophy—RV free wall = 8 mm; (**F**) RV severe systolic dysfunction (TAPSE = 13 mm) (**G**) LA speckle-tracking showing significantly decreased pump function (white arrow) and even more severe reservoir function (red arrow), with the absence of conduit function. RWT, relative wall thickness, E = early filling velocity; A = late atrial filling velocity; S’, systolic TDI velocity; E’, early diastolic TDI velocity; A’, late diastolic TDI velocity; LA, left atrium; LV, left ventricular; RV, right ventricular; TAPSE, tricuspid annular plane systolic excursion; STE, speckle tracking echocardiography.

The typical CA phenotype is characterized by increased LV wall thickness (LVWT) (>12 mm), small LV, biatrial dilation, and restrictive diastolic filling. LVEF declines with the progression of the disease. Diastolic dysfunction is attributed to amyloid deposition and inflammation and progresses from impaired relaxation to a restrictive pattern in advanced stages [9] (Figure 2).

Other echocardiographic signs suggestive of CA include pericardial effusion, right ventricular (RV) hypertrophy, and atrial septal and valve thickening [9]. RV dilation may occur late in the disease and carries a poor prognosis. None of the previously cited parameters are pathognomonic for ATTR-CM [56]. ATTRwt seems to have greater LVWT and LV mass, by comparison with ATTRm and AL-CA, probably due to a longer time of fibrils accumulation [57,58]. Structural changes in CA are due mainly to interstitial amyloid infiltration, with amyloid deposits determining a speckled pattern of the myocardium (“granular sparkling”), which can be diffuse or sometimes localized in specific segments of the ventricular wall (such as the ventricular septum or the posterior wall), with apical sparing [9,59,60,61].

RV involvement occurs after LV. TAPSE <14 mm independently predicted the risk of acute HF, death, and heart transplantation [62,63]. Damy T et al., in a group of 266 patients with CA, found that pericardial effusion, even in small amounts, is a strong predictor of death, without any difference regarding subtype [40].

***New Echocardiographic Techniques***

**Figure 3:**
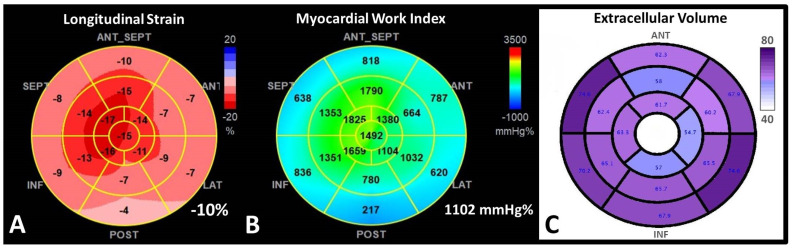
**Apical sparing pattern in ATTR cardiac amyloidosis.** (**A**) Longitudinal deformation bull’s eye plot of the left ventricle by speckle−tracking−echocardiography in a 46-year-old patient with ATTR-CM with significantly decreased global LS of −10%, with longitudinal deformation impaired at the basal, midventricular segments, and relatively preserved at the apex, in an “apical sparing” pattern. (**B**) The corresponding myocardial work index (MWI) bull’s eye plot, confirming a similar apical sparing pattern, with preserved MWI in the apical segments; (**C**) Extracellular volume (ECV) bull’s eye plot evaluated by cardiac magnetic resonance, using a 16-segment model, displaying a progressive decrease in the ECV from base to apex, explaining the substrate of apical sparing deformation pattern.

1. *Tissue Doppler Imaging (TDI).* The longitudinal myocardial velocities recorded at the level of the lateral and septal mitral annulus in CA are significantly lower than in patients with true hypertrophy, despite normal LVEF in both categories [59]. TDI in CA demonstrates that longitudinal ventricular contraction is impaired well before deterioration of the LVEF and that longitudinal dysfunction precedes the onset of HF [64]. A decrease in RV free wall systolic velocity has been also recorded [62,65]. However, the prognostic value of the TDI in CA is not clearly established.
2. *2D Speckle Tracking Echocardiography (STE).* STE is considered nowadays a more feasible and robust technique than TDI strain. In CA with preserved LVEF, strain and strain rate determined through STE are reduced compared to healthy people, unlike TDI velocities, which remain almost normal in the early stages of the disease [66]. The relative apical-sparing pattern of the global longitudinal strain (**LS**) is considered highly suggestive of CA [56,63] (Figure 3).

Pagourelias et al. demonstrated that in patients with LV hypertrophy, the LVEF to LS ratio has the best accuracy to detect CA, with very good sensitivity and specificity, independent of CA subtype [67]. The ratio of average apical LS/ (average basal LS + average mid-LS), named relative apical LS index, is useful for the detection of CA under LVH [68]. Moreover, a cut-off value of septal apical to basal LS ratio > 2.1 differentiated CA from other causes of LVH [69].

A new model incorporating relative LVWT, E/E’ ratio, LS, and TAPSE was found to have the greatest diagnostic performance in AL-CA, whereas the addition of septal apical–to–base ratio yielded the best diagnostic accuracy in the increased heart wall thickness group, including the confirmed ATTR patients [55]. It is worth mentioning that ***red flag echocardiographic signs*** emphasized by the current guidelines are detected in all forms of CA and include the following: granular sparkling of the myocardium, reduced LS with an apical sparing pattern, increased LV and RVWT, increased valve thickness, and pericardial effusion [9].

Moreover, conventional parameters such as decreased LVEF [10], greater LVWT [8], restrictive diastolic dysfunction, increased E/E’ ratio, and RV involvement [24] had important prognostic value [3]. LS by STE has an incremental prognostic value, connected with poor survival rate in both types of CA. A study conducted by Lee et al. has demonstrated that patients with early CA (normal LVEF, preserved E/E’ and high LS) had an apical sparing pattern, but they had a lower relative apical LS index than patients with advanced CA [70,71]. In the ATTR-CM, an apex-to-base RV strain gradient was noted, with relative apical sparring, similarly to LV findings [72]. Baseline RV free wall LS determined by STE was independently associated with cardiovascular hospitalizations and all-cause mortality, at one year. RV free wall strain decreased significantly in CA during follow-up, whereas other RV functional parameters remained stable [72]. Chacko et al. aimed to characterize echocardiographic phenotype in 1240 patients with ATTR-CM (766 with ATTRwt and 474 with ATTRm) who were followed up for survival for ten years. At diagnosis, patients with V122Ih ATTRm had the most severe LV dysfunction, and patients with T60AhATTRm the least, while patients with ATTRwt had intermediate features. E/E’, Stroke volume index, right atrial area index, and LS were all predictors of mortality. Moreover, severe AS was independently associated with significantly shorter survival [73].

Recently, LS and left atrial (LA) dysfunction were found to be independent and powerful prognostic markers in patients with CA [74]. In patients with AL CA, LS less than −14.8% has been shown to independently predict all-cause mortality [10]. However, data in ATTR-CM are scarce.

3. *Myocardial work analysis derived from STE*. Myocardial Work (MW) is a novel non-invasive echocardiographic technique for myocardial performance assessment, derived from LV pressure-strain loop analysis. By integration of afterload, MW analysis might have a superior benefit in the evaluation of the prognosis of patients. MW is abnormal in patients with many forms of LVH: hypertrophic cardiomyopathy, hypertensive cardiomyopathy, AS [75]. New evidence demonstrated a potential role for new MW analysis, a novel STE measure of LV systolic function, which may be more sensitive than LS in the diagnosis and prognosis of CA (Figure 3). Both global work index (GWI) and global efficiency (GWE), showed a good correlation with NT-proBNP, eGFR, TpI, and peak oxygen consumption [75,76,77]. These indexes might be better used to assess the efficiency of the treatment than LS or LVEF, because loading conditions are variable over time [75,76]. Furthermore, MW indices seem to predict all-cause mortality in CA better than LVEF.

All-cause mortality was best predicted by GWI < 937 mmHg/% and GWE <89% [75]. Moreover, the optimal cut-off points for MWI and LS to discriminate patients in NYHA functional class III from those in NYHA functional class II were MWI < 987 mm Hg% and LS < 10.4% [77]. In addition to global MW analysis, segmental analysis of MW revealed an apical sparing pattern in patients with CA, leading to significant alterations in the average apical-to-basal segmental ratios. This ratio was found a significant predictor of all-cause mortality after adjustment for LS, NTproBNP, TpT, and creatinine levels [77].

The high prevalence of HFpEF in ATTR-CM limits the utility of LVEF for risk stratification and emphasizes the importance of myocardial deformation and work evaluation for outcome prediction in CA patients. Apical-to-basal segmental work ratio combined with GWI might represent powerful predictors of MACE and mortality in ATTR-CM patients that need to be validated in larger studies [77]. It should be used to better assess the severity of the disease and to better select candidates for therapeutic intervention.

4. *Left atrial strain analysis by STE*. Structural and functional assessment of the LA is an important tool, due to the fact that LA dimensions and functions are independent predictors of survival in HF, especially in HFpEF. Recently, LA functional assessment has become more accurate using STE, from which myocardial strain (S) and strain rate (SR) can be evaluated during different phases of the cardiac cycle [78]. LA function has been assessed comparatively in patients with different types of CA (AL, ATTRm, and ATTRwt) and matched healthy volunteers. Nocioka et al. showed that all LA functions were severely decreased in CA, expressed by conventional LA volumes and functions, and also by S and SR parameters. Among the different CA subtypes, LA reservoir strain (LAr) and LA active emptying fraction were worse in ATTRwt than AL and ATTRm [79]. LA reservoir and pump function are significantly impaired in both ATTR-CM and hypertrophic cardiomyopathy (HCM) patients compared with controls, irrespective of LA volume and LVEF, more severe in CA, mainly determined by the LA wall infiltration [80].

Moreover, current evidence reported absent atrial contractility in CA, even in sinus rhythm patients, leading to a high incidence of thrombus formation [63]. However, there is no evidence to support anticoagulation for patients in sinus rhythm, yet accurate evaluation of all LA functions through STE represents a useful tool to identify patients with higher thromboembolic risk (Figure 2). In CA patients, atrial fibrillation might occur in the absence of LA enlargement. Henein et al. aimed to assess the relationship between LA size and function and their relationship with atrial arrhythmia in patients with ATTRm. In addition to conventional measurements and LS, LAr strain and strain rate were obtained. ATTR patients with increased LVWT were compared with patients with HCM and healthy volunteers. Atrial deformation during atrial systole was significantly reduced in ATTR patients with increased LVWT, independent of LA size, and in contrast to HCM. LA strain rate, during atrial systole, was the only independent predictor of atrial arrhythmia [78].

In summary, echocardiographic assessment of LA function by STE should be performed routinely, both for the diagnosis, for thrombotic risk prediction, as well as for monitoring of patients with CA.

5. *3D Echocardiography (3DE).* 3DE and 3D speckle tracking echocardiography (3DSTE) are increasingly used for characterizing cardiac structure and function, and they have been also used for a more accurate assessment of CA. Deformation and rotational 3DSTE parameters seem to be able to differentiate CA patients from patients with other forms of LVH. Basal rotational strain determined through 3DSTE is significantly lower than apical rotational strain in CA compared to HCM [81]. However, 3DE is not recommended by the current guideline [9], and more clinical studies are needed in order to implement 3DE as a routine diagnostic tool for CA.

A common evaluation and follow-up scheme, included in the guidelines, consists of yearly visits with complete 2DE in both ATTR-CM patients and ATTRm asymptomatic genetic carriers [9].

The present guidelines proposed an echocardiographic score-based diagnosis for CA [9]. This score system consists of an association between:

**I. Unexplained LV thickness (>12 mm);**

AND

**II. more than 2 criteria from:**

- Diastolic dysfunction higher than grade 2;
- Reduced TDI velocities (<5 cm/s);
- LS < −15%.

OR

**III. Multiparametric echocardiographic score > 8 points**:

- Relative LVWT (IVS + PWT)/LVEDD > 0.6—3 points
- E/E’ ratio > 11—1 point
- TAPSE < 19 mm—2 points
- LS < −13%—1 point
- Systolic longitudinal strain apex to base ratio > 2.9—3 points

However, current evidence does not support the guidelines proposed parameters as being useful for treatment monitoring.

## 5. Cardiac Magnetic Resonance

CMR has emerged as a useful tool in the diagnosis, risk stratification, and prognosis of CA [9,82]. CMR is considered the gold standard for the evaluation of the structure, and global and regional function [64,83]. CMR can visualize, with late gadolinium enhancement (**LGE**), and measure, with T1 and T2 mapping, the continuum of cardiac amyloid deposition [83,84,85].

**Figure 4:**
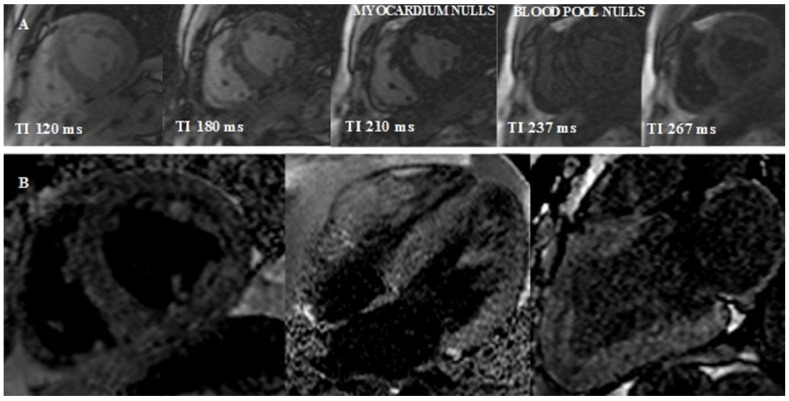
Reversed Look–Locker inversion time nulling pattern in cardiac amyloidosis. (**A**) Sequential Look–Locker inversion time (TI) mid-ventricular short-axis images are shown at increasing inversion times from left to right. Blood pool nulls after myocardium, which is an abnormal (reversed) nulling pattern. (**B**) Midventricular short-axis, 4-chamber, and 2-chamber LGE images demonstrate a dark blood pool and difficulty nulling the myocardium.

1. *Early gadolinium enhancement* (*EGE*) images are usually acquired in the first 3 to 5 min after gadolinium contrast agent (GCA) administration. This agent is able to penetrate vascular structures. In CA there is evidence of microvascular coronary obstruction and severe endothelial dysfunction from histological studies. Therefore, EGE imaging is useful for the detection of microvascular obstruction (MVO) [63].
2. *Look-Locker TI scout*. The optimal inversion time to null the normal myocardial signal is determined using a Look–Locker TI scout sequence acquired in the short-axis at the mid-ventricular level ∼5 min after the administration of contrast. The *pattern* of nulling is classified as normal if the blood pool is nulled before the myocardium. In CA the pattern is reversed, blood pool nulling being coincident with or after myocardial nulling (Figure 4).

3. *Late gadolinium enhancement* (*LGE)* can be seen in three possible patterns: no LGE, sub-endocardial -, and transmural enhancement [83]. Sub-endocardial LGE appears to be more prevalent in AL-CA, whereas transmural LGE is more prevalent in ATTR-CM. In addition, RV and LA LGE were found to be more prevalent in patients with ATTR [8,9,10,11,12,13,14,15,16,17,18,19,20,21,22,23,24,25,26,27,28,29,30,31,32,33,34,35,36,37,38,39,40,41,42,43,44,45,46,47,48,49,50,51,52,53,54,55,56,57,58,59,60,61,62,63,64,65,66,67,68,69,70,71,72,73,74,75,76,77,78,79,80,81,82,83]. Transmural LGE carries higher mortality rates compared with subendocardial LGE [83,84,85]. The mechanism of LGE in CA is due to infiltration of the amyloid protein and fibrosis caused by ischemia due to capillary obstruction by amyloid deposits [63].
4. *T1 mapping*. The use of GCA is relatively contraindicated in severe renal failure. *Native T1 mapping*, before the administration of GCA, can overcome this limitation, as it measures direct quantitative signals from the myocardium [86,87,88]. Post administration of GCA the myocardial extracellular volume (*ECV*) can be calculated (Figure 3). Native myocardial T1 showed a high diagnostic accuracy to discriminate CA (AUC = 0.93). T1 mapping measures myocardial amyloid load and myocyte response to infiltration, allowing monitoring and eventual change of therapy, even when cardiac function is normal.

Baggioano et al. proposed an algorithm with reliable cut-offs for native myocardial T1 that exclude or confirm the diagnosis of CA and restrict administration of GCA only to patients with an intermediate probability of CA. Myocardial native T1 < 1036 msec excludes CA with very high diagnostic accuracy (NPV 98%). In patients with native myocardial T1 > 1164 msec, CA can be diagnosed with very high diagnostic accuracy (PPV 98%). When T1 ranges between 1036 and 1164 msec, GCA should be considered, the probability of CA being intermediate [89]. Using this algorithm, the diagnosis of CA could be obtained in 42% of patients with suspected CA without the need for GCA.

In patients with intermediate probability based on the native T1, an ECV cut-off of 37% was associated with a very high diagnostic accuracy (AUC 0.976). All these values are obtained with magnetic fields of 1.5 T. There are no data on 3T machines. These results support the role of ECV as a diagnostic marker in patients with suspected CA undergoing a CMR that includes the administration of GCA. ECV has advantages over the LGE evaluation, as it has the ability to quantify the expansion of extracellular space. Multiple studies showed that ECV and T1 reliably distinguish CA from other diseases [89,90,91]. Both native myocardial T1 and ECV have been extensively validated in CA as surrogate markers of infiltration. Moreover, ECV and native T1 are significantly elevated even in patients with CA and normal LGE imaging, being useful as early disease markers [86,87]. ECV was found to be an independent predictor of mortality [87].

ECV is correlated well with 99mTc-DPD scintigraphy [87,88]. Fontana et al. showed that native T1 and ECV predicted death, but only ECV remained an independent predictor of mortality after adjustment for age, NTproBNP, LVEF, E/E’, LV mass index, DPD grade, and LGE, suggesting that ECV is a more robust marker in ATTR-CM [84,87]. Moreover, they demonstrated a good correlation between ECV and native T1 for a low level of infiltration, but a very poor correlation when the amyloid burden is moderate or severe [87]. Both myocardial T1 and ECV correlated with higher Perugini grades on CS [87]. These differences are generated by different biological information provided by native T1 and ECV. CA is characterized by different degrees of amyloid infiltration, myocardial edema, inflammation, and differential myocyte response with myocyte hypertrophy. ECV measurements enable us to collect the signal from the extracellular space, whereas native myocardial T1 provides a composite signal from the intra and extracellular spaces, potentially influenced by other pathophysiological mechanisms beyond amyloid load. Native myocardial T1 will therefore be significantly raised by the presence of extra and/or intracellular myocardial edema, whereas ECV will be elevated when there is extracellular edema [92,93].

5. *T2 imaging* is a well-established non-*contrast* technique that quantifies myocardial edema.

The most recent study in 100 AL and 186 ATTR CM patients showed a higher mean T2, compared with healthy volunteers. A higher T2 was correlated with myocardial edema on histological examination being associated with worse systolic function [93]. In this study, T2 predicted mortality only in AL amyloidosis even after adjusting for ECV and NTproBNP. This suggests that edema is a less important factor in the pathogeny of ATTR-CM than AL [93].

**Figure 5:**
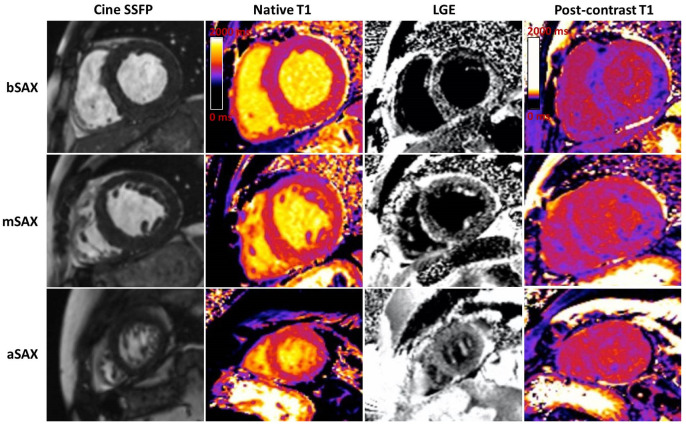
Typical cardiac magnetic resonance findings in a patient with ATTR cardiac amyloidosis. Short axis views at basal (bSAX), midventricular (mSAX), and apical (aSAX) levels showing concentric left ventricle hypertrophy in SSFP cine images (the first column), with diffuse elevation in native T1 mapping (the second column), transmural hyperenhancement, more pronounced at the basal and midventricular levels, with a dark blood pool in LGE images (the third column) and diffuse reduction in post-contrast T1 mapping (forth column). SSFP: Steady-state free precession; LGE: late gadolinium enhancement.

To summarize, typical CMR findings in ATTR-CM are as follows (Figure 5):

- LVH, restrictive LV pattern (preserved LVEF, non-dilated ventricles, enlarged atria);
- Reduced LV indexed stroke volume;
- Atrial septal hypertrophy;
- Mild pericardial effusions;
- Abnormal nulling time for the myocardium;
- LGE from sub-endocardial to transmural myocardium;
- RV involvement with hypertrophy and in advanced stages with RV LE;
- Atrial LGE is a strong clue, and is associated with atrial contractile dysfunction;
- Significantly increased native T1 time and ECV compared to other causes of LVH (ECV > 40%) [9].

However, current recommendations emphasize the idea that CMR cannot reliably differentiate subtypes of CA [9]. There are limited data about CMR utility in monitoring response to therapy. ECV and LV mass were identified in very small studies as potential parameters useful to be monitored, after 12 months of treatment with new therapy, such as tafamidis, inotersen, and patisiran [83,84,85,86].

## 6. Nuclear Imaging Role in ATTR-CM

**CARDIAC SCINTIGRAPHY**. Neither echocardiography nor CMR provides information regarding the type of CA. Currently, cardiac scintigraphy (CS) is considered the most accurate imaging technique that contributes the most to the diagnosis of ATTR-CM. Therefore, CS is at the center of the ATTR-CM diagnostic algorithm [9,94]. CS with ^99m^Tc-pyrophosphate (^99m^Tc-PYP), ^99m^Tc-3,3-diphosphono-1,2-propanodicarboxylic acid (^99m^Tc-DPD), or 99mTc-hydroxymethylene diphosphonate (HMDP) has a high level of diagnostic accuracy in differentiating CA subtypes, when scintigraphy shows grade two or three myocardial uptake [9]. It is worth mentioning that even if there is no direct comparison between the mentioned radiotracers, CS with ^99m^Tc-HMDP has the lowest sensitivity in detecting CA [9].

Biopsies from ATTR-CM patients have higher microcalcification density compared to AL-CA, providing a possible explanation for higher uptake in the ATTR subgroup [95,96]. This binding allows a semiquantitative scoring and a quantitative (heart to contralateral ratio) evaluation of amyloid burden [94]. A large multi-center study including 1217 patients with suspected CA demonstrated that CS with grade two or three myocardial radiotracer uptake had a 100% specificity and positive predictive value for ATTR-CM in the absence of monoclonal protein in serum or urine [97]. Therefore, guidelines clearly specify that clonal dyscrasia should be excluded: serum-free light chain (FLC) assay, serum (SPIE), and urine (UPIE) protein electrophoresis with immunofixation [9]. All three hematological tests combined have a sensitivity of 99% for identifying abnormal precursor in AL amyloidosis [9].

**Figure 6:**
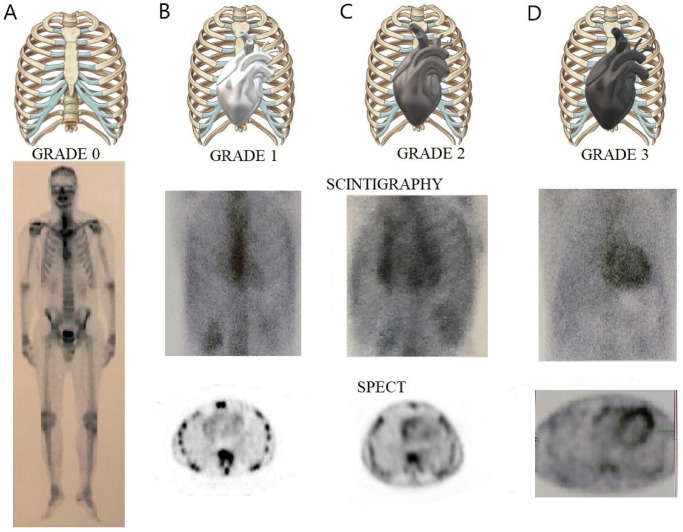
**Cardiac scintigraphy with ^99m^Tc-PYP in ATTR cardiac amyloidosis**. Upper panel illustrating with 3D chest impression Perugini grading scale of myocardial uptake. Lower panel illustrating the presence of a heart silhouette on chest planar imaging (middle row), and the radionuclide distribution at the level of the myocardial walls on SPECT imaging (bottom row) in varying degrees, from left to right. (**A**) Perugini score 0—no cardiac uptake. (**B**) Perugini score 1—low cardiac uptake below the bone uptake. (**C**) Perugini score 2—moderate cardiac uptake similar to the bone tissue. (**D**) Perugini score 3—high cardiac uptake greater than rib uptake, with biventricular involvement.

On planar images, cardiac involvement can be evaluated using a visual score (**Perugini score**) [8] that compares the degree of cardiac and bone uptake (0 = absent; 1 = less than bone; 2 = equal to bone; and 3 = high cardiac uptake greater than bone) (Figure 6). Grade 2 or higher is considered highly specific for the diagnosis of ATTR-CM, but monoclonal protein should be excluded [8,9]. However, the Perugini score had no prognostic significance in patients with CA [9,98].

A recent systematic review also confirmed the accuracy of scintigraphy in the diagnosis of ATTR-CM, having both sensitivity as well as specificity above 90% [99]. Moreover, CS may be of particular interest in ATTR patients who have an unrelated monoclonal gammopathy of unknown significance (MGUS), a condition associated with ATTRwt, because the presence of MGUS may generate an incorrect diagnosis of AL-CA [100].

In order to prevent misdiagnoses, scintigraphy should always be accompanied by a single-photon emission computed tomography (SPECT) evaluation. It will confirm whether cardiac uptake corresponds to the myocardium or not [9,101]. By combining CS and SPECT, Hutt et al. developed a new grading system: Grade 0 = no visible myocardial uptake on CS and cardiac SPECT images; Grade 1 = cardiac uptake seen only by SPECT, or minimal cardiac uptake (less intense than bones) on the CS; Grade 2 = moderate cardiac uptake, greater in intensity than the bone uptake, with apparent reduction of the latter on CS imaging; Grade 3 = intense cardiac uptake with little or no bone uptake visualized on CS imaging; Grade 4 = intense uptake partly or completely obscuring cardiac uptake on planar imaging [102].

Initially, a 3 h protocol was used for CS. Recently, it has been shown that a 1 h protocol is comparable to the 3 h protocol [102], with a 98% sensitivity and a 96% specificity for CS and SPECT, identical between protocols. The advantage of the 1 h protocol is high: it reduces cost and time, without compromising the diagnostic accuracy [103,104].

CS is very useful for the early diagnosis of CA because it detects myocardial infiltration before the echocardiographic abnormalities [93,94]. Grade 0 compared to grades 1, 2, and 3 showed a better survival rate, but without any differences between the non-zero degrees. A heart to contralateral ratio > 1.5 had a sensitivity of 97% and a specificity of 100% for the diagnosis of ATTR-CM, and a ratio > 1.6 was associated with significantly worse survival [94]. An apical-sparing pattern on CS images was described by Sperry et al., comparable with the basal to apex gradient from the Bull’s eye map at 2DSTE. Patients with diffuse infiltration had more apical uptake and worse survival. A new nuclear imaging marker, apical sparing ratio (ASR), which is calculated by adding base and mid-segment values and dividing the total by apical counts, has also been shown to predict survival better than echo-derived LS. An ASR > 2.75 is associated with a better prognosis in patients with ATTR CM [105].

New markers derived from 99mTc-DPD scintigraphy used for the quantitative assessment of the amyloid burden are the ratio between radiotracer in the heart and retention in other body parts, heart/whole-body ratio (H/WB), and heart/contralateral lung ratios (H/CL) [101]. ATTR-CM has a higher H/WB ratio than AL-CA, correlated with cardiac events in patients with hereditary ATTR-CM [101]. A recent study has found that the amyloid loading SPECT has correlated with LS, troponin, and NTproBNP [106].

There are some rare situations of false positive and false negative cardiac uptake, which should not be forgotten when reporting scintigraphy results. False negative results are found in mild ATTR-CM, specific mutations in ATTR (Phe64Leu, Ser97yr), and premature/delayed acquisition, whereas false positive results occur in severe cardiac dysfunction (blood pool), valvular/annular calcification, AL amyloidosis, AApoAI and AApoAII, ApoAIV, Aβ2M amyloidosis, rib fractures, recent myocardial infarction (<4 weeks), and hydroxychloroquine cardiac toxicity [9].

In summary, CS provides several advantages: early diagnosis and the ability to distinguish the different types of CA, with the disadvantage that it requires, equally to SPECT, exposure to ionizing radiation. Data about the role of scintigraphy in longitudinal monitoring after new types of treatment initiation are not yet available.

**POSITRON EMISSION TOMOGRAPHY (PET)** is another promising imaging technique that may help distinguish between amyloidosis subtypes [9,101,104]. A higher uptake has been reported in patients with CA, by comparison to other cardiomyopathies. The potential benefits of PET-based radiotracers include better sensitivity for AL-CA diagnosis and more accurate monitoring of the response to treatment. Different tracers were evaluated such as 18F-florbetapir, 18F-florbetaben, and 11C-Pittsburgh Compound-B [107,108]. 11cPiB PET was found to have 100% diagnostic accuracy for AL-CA. Positive 11C-PiB uptake on PET and negative CS was seen in all patients with AL-CA, whereas positive CS combined with negative 11C-PIB uptake was reported in all patients with ATTRwt [109]. Moreover, increased 11c-PiB uptake has been associated with negative outcomes [110].

As effective treatment options for ATTR-CM are available now, a critical need has emerged for an imaging test to monitor disease activity by serial imaging. All current imaging approaches have limitations in this regard, including CS and CMR. With its superior ability to quantify tracer uptake, PET-based tracers are being actively investigated for serial imaging [15,19,33].

**COMPUTED TOMOGRAPHY.** Improvements in CT imaging technique have extended the utility of cardiac CT to myocardial characterization via late iodine enhancement (LIE) imaging as well as ECV quantification [111]. ECV assessed by CT correlates well with the ECV obtained by CMR [112]. The aortic valve calcium score assessed by non-contrast CT helps to grade AS severity, even in CA associated with AS [113]. Supplementarily, the assessment of myocardial ECV during SAVR or TAVI planning CT can discover concomitant CA in patients with severe AS [114]. Moreover, CT scans can identify lung and respiratory tract involvement (nodular pulmonary amyloidosis, diffuse alveolar-septal, tracheobronchial, or pleural amyloidosis) [115].

## 7. ATTR-CM in Aortic Stenosis Has a Particular Need for Screening and Treatment

There is increasing interest in the coexistence of CA and AS because of the observed age-correlated prevalence and higher mortality in these groups of patients compared to AS alone, which might be influenced by the new management options, including TAVI and targeted new therapies for ATTR-CM [9,15]. Recent data support TAVI procedures in AS-CA patients, showing a worse prognosis when AS was left untreated [2,9]. A 56% 1-year all-cause mortality was reported in patients with AS and ATTR-CM compared to 20% for isolated AS [15].

The most common clinical scenario for a patient with ATTR-CM in AS is a male patient >65 years old or a female patient >70 years old with HF or the presence of several signs/symptoms that are listed as “red flags” in Table 1. In addition, echocardiographic changes particularly found in AS with ATTR-CM are as follows [3,20,21,116]:

▪ LVH (interventricular septum >18 mm) with infiltrative features (Myocardial granular sparkling), increased thickness of atrioventricular valves, interatrial septum >2 mm, and RV free wall ≥5 mm with RV dysfunction: tricuspid S’ < 9 cm/s and TAPSE <14 mm)
▪ Small LV cavity with reduced stroke volume (SVi < 30 mL/min/m^2^)
▪ Bi-atrial enlargement and small A wave on mitral inflow Doppler;
▪ Restrictive diastolic pattern with signs of high LVFP in advanced disease;
▪ Pericardial effusion
▪ Early impaired longitudinal strain (LS < −12%, mitral S’ ≤ 6 cm/s);
▪ Apical sparing with normal LVEF (this hallmark appearance on the “bull’s eye” plot on STE is not a typical finding in severe AS patients, probably secondary to diffuse LV remodeling in these settings)

Compared with patients without ATTR-CM, ATTR-CM and AS patients had a thicker interventricular septum, higher LV mass index, and lower SVi. ATTR-CM patients with AS had advanced diastolic dysfunction with a higher E/A ratio and impairment in systolic function with lower LVEF. While ATTR-CM patients had more impaired LS (−12 vs. −16%), the relative apical longitudinal sparing pattern was the same regardless of ATTR-CM diagnosis. Average S’ < 6 cm/s best predicted ATTR-CM in AS in multivariable logistic regression (100% sensitivity for predicting a positive CS) [117]. Both AS and CA share several clinical and imaging elements, such as LVH, diastolic dysfunction, and impaired LS. Therefore, diagnosis of CA in AS patients may be very challenging and requires more specific “red flags” for an accurate assessment. Nitsche et al. [2] developed a score, the RAISE score, to help predict the presence of CA in AS patients. The following parameters were included:

▪ History of carpal tunnel syndrome (3 points)
▪ Right bundle branch block (2 points)
▪ Sokolow/Lyon index < 1.9 mV (1 point)
▪ High sensitivity troponin level >20 ng/mL (1 point)
▪ E/A ratio > 1.4 (1 point)
▪ Age ≥ 85 years (1 point)

Further studies are needed to validate this score, but it might become a useful tool to raise suspicion and orientate the following steps. A score ≥ 2 requires confirmation for CA in AS using CS and hematological analysis [116].

CMR evaluation revealed similar findings as in the general population with ATTR-CM. However, it has been reported that CMR might be a poor diagnostic tool in patients with ATTR-CM and AS, one study showing specific CMR features of CA only in two out of six confirmed cases on biopsy during SAVR [17].

The era of emerging therapies for ATTR (tafamidis, patisiran, inotersen) [82,118,119,120] presents new opportunities to study disease modulation in patients with concomitant ATTR-CM and severe AS. Whether this therapy could improve HF hospitalization and mortality in ATTR-CM patients with AS following TAVI is an area of future study for patients with early disease. Prospective studies are needed to determine the potential role and appropriate timing of interventional and pharmacologic management strategies for these patients with the goal of not only improving mortality but also morbidity associated with concomitant disease.

## 8. Conventional and New Added Biomarkers in ATTR-CM

In CA, biomarkers might play an important role at all levels of the algorithm, from screening to diagnosis, prognosis, risk stratification, and monitoring of response to therapy. Once the diagnosis is confirmed, Tp and NT-proBNP were found to be the strongest predictors of survival in patients with ATTR [121,122]. Both are often elevated early in patients with ATTR-CM, out of proportion to the degree of HF [7]. However, troponin and NT-proBNP have low sensitivity and specificity in the initial diagnosis of ATTR-CM [3]. Currently, they are used in several staging systems in order to facilitate prognosis, but there are limited data about their utility in assessing disease progression, therapy-decision making, and follow-up [9]. Following the staging systems validated for prognosis in AL amyloidosis, two different biomarker-based staging systems have been proposed for ATTR-CM [121,122].

In a cohort of patients diagnosed with ATTRwt cardiomyopathy, Grogan et al. assessed NT-proBNP and high-sensitivity troponin T (hsTnT) as predictors of overall survival (OS). Based on their findings, Grogan et al. proposed a staging system, similar to the biomarker system defined for AL amyloidosis that can provide prognostic information and risk stratification. The ATTRwt staging system used a higher NT-proBNP cut-off value >3000 pg/mL, compared to the cut-off point > 1800 pg/mL used in AL amyloidosis, and a cut-off value of ≥ 0.05 ng/mL for hsTnT [121]. Grogan et al. defined stages I, II, and III based on a median OS of 66, 40, and 20 months, respectively [123,124]. Gillmore et al. developed another universally applicable staging system, similar to AL-CA, based on NT-proBNP and estimated glomerular filtration rate (eGFR), which provided risk stratification of patients with both ATTRwt and ATTRm [125]. The cut-off values were NT-proBNP >3000 pg/mL and eGFR > 45 mL/min/1.73 m^2^ [9]. The reported OS for stages I, II, and III were 62, 47, and 24 months, respectively [122]. The two available staging systems encompass easily accessible biomarkers, with the potential to provide stratification for clinical and research purposes, enabling the enrolment of patients in further clinical trials of novel therapies in an era of rapidly emerging therapeutic interventions [121,124,125]. Both staging systems were compared in terms of predictive performance. However, they identified concordance in classification only for 70% of patients [123]. The combination of NT-proBNP and eGFR offers a more robust prognosis evaluation and a better stratification of patients in three subsets, with different survival and risk for all-cause mortality, compared to the biomarker combination of NT-proBNP and Tp [123]. Nonetheless, staging systems for ATTR-CM have not been well validated and at the moment there is no single system in consistent use [9].

Takashio et al. found that hs-cTnT levels could discriminate between CA and other causes of cardiac hypertrophy, with higher levels of troponin in CA [126]. All findings suggest that Tp could represent a nonspecific marker of chronic cardiac damage, with less particular serum dynamism across all stages of disease severity, while NT-proBNP and eGFR might represent more vigorous markers in early cardiac involvement identification and prognosis stratification [123].

The emergence of novel pharmacotherapies for ATTR-CM imposes the need to explore the utility of ***new biomarkers*** as early diagnostic tools and preclinical markers for screening populations at risk [118,127,128]. At present, TTR levels and RBP4 have been explored as potential markers of preclinical ATTR-CM [127].

Recent evidence has shown that **TTR** has been associated with various biological processes that are associated with oxidant and antioxidant properties and has implications for potential disease-specific biomarkers or therapeutic targets [129]. TTR values correlate with the degree of TTR instability and are associated with survival in ATTR [127]. Moreover, gene silencing therapeutic agents reduce serum TTR levels, acting as a marker of therapeutic efficacy [127]. In patients treated with TTR stabilizers, such as tafamidis and diflunisal, higher levels of TTR compared to non-treated patients were observed [130]. Furthermore, Hanson et al. showed that lower TTR concentration correlated with reduced survival as an independent predictor of prognosis, in addition to other parameters of cardiac functional status [122]. Longitudinal assessment confirmed that decreasing TTR concentration is consistent with worsening cardiac function, suggesting that TTR levels could be an independent predictor of outcomes in ATTR [122].

Lower levels of **RBP4** appear to be aligned with serum TTR levels [128]. In a small cohort comprising 47 patients with ATTRwt and 27 patients with V122I-ATTRm, RBP4 concentration was lower compared with healthy subjects and correlated with TTR level [131]. This study suggested that RBP4 is associated with pathogenic TTR mutations, emphasizing RBP4 as a sensitive marker for ATTR screening [127]. A clinical prediction model based on RBP4 levels, LVEF, LVH, and mean limb lead voltage, was suggested as having the ability to identify ATTR-CM, and thus trigger further diagnostic investigations [12]. Additionally, RBP4 concentration might be useful as an initial screening test, being associated with ATTRm independent of potentially confounding variables [12].

It has been well understood that toxic TTR oligomers preferentially deposit in the extracellular matrix of cardiac and neuronal cells resulting in significant tissue injury and **progressive inflammatory response** [129]. Moreover, there is a strong correlation between TTR levels and the generation of superoxide radicals (ROS) and nitrate and nitrite ions (RNS), which enhances the need for the determination of TTR oligomer levels in order to assess **the extent of oxidative stress** [129].

Areas of research interest in CA include the evaluation of novel biomarkers such as **matrix metalloproteinases (MMP) and tissue inhibitors of metalloproteinases (TIMP),** underscoring their potential in differentiating between AL and ATTR-CM [121]. Tanaka et al. found that patients with AL-CA present higher levels of MMP-2 and TIMP-1, MMP-2/TIMP-2, BNP, and TpI compared to patients with ATTR-CM [132].

Current data suggest that inflammation, oxidative stress, reduced nitric oxide (NO) bioavailability, endothelial dysfunction, and myocardial remodeling and fibrosis are involved in the pathogenesis of ATTR-CM [9]. Thereupon, the use of new biomarkers might provide input from different pathophysiological pathways that could offer incremental data in the diagnosis, prognosis, and identification of patients with a high risk of ATTR-CM progression [9,133]. Novel knowledge on the alterations in immune response in patients with ATTR suggests a significant role of inflammation in ATTR progression that can add incremental value to the understanding of the natural history of subclinical cardiac amyloidosis [134]. Increased levels of six inflammatory cytokines (TNF-α, Il-1β, IL-33, IFN-β and IL-10) have been identified in patients with symptomatic ATTRm, while elevated levels of IL-33, IL-1β, and IL-10 were present in asymptomatic patients, suggesting that inflammation might occur before fibril deposition [134]. Moreover, these novel findings suggest that related to TNF-α, cytokine levels positively correlate with disease progression, indicating the implication of this cytokine in ATTRm pathogenesis [134]. Systemic inflammation determines coronary microvascular endothelium to increase the production of reactive oxygen species, reducing NO bioavailability and generating a reduction in cGMP availability, thus causing remodeling, impaired relaxation, and myocardial stiffness [7,9,134]. Several specific biochemical markers from this chain, relating to myocyte stress, inflammation, endothelial dysfunction, NO bioavailability, thrombosis-haemorrhagic risk, and extracellular matrix remodeling, appeared to be promising diagnostic and prognostic tools in patients with ATTR-CA.

However, data about systemic inflammation biomarkers in ATTR-CA are scarce, compared to the well-defined status in risk stratification of cardiovascular events in HF for biomarkers such as soluble suppression of tumorigenicity 2 (sST2), IFN, TNF, GDF15, pentraxin-3, and lipocalin-2/NGAL [13,135,136]. An observational case-controlled study that compared proteomes of patients with ATTRm and healthy controls, in a subset of patients enrolled in the APOLLO study, found that neurofilament light chain could serve as a biomarker of nerve damage and polyneuropathy in ATTRm, enabling early diagnosis and monitoring of disease progression [137].

Recently, Misumi et al. identified that fibroblasts and macrophages play a decisive role in the degradation of aggregated TTR [138]. Heart tissue-resident macrophages present an inhibitory phenotype [139]. Quantitative reduction of tissue-resident inhibitory macrophages triggers TTR deposition in heart tissue, underlying the involvement of inflammation in the pathogenesis of the disease [139]. The inflammatory component of the ATTR pathogenesis is further supported by studies oriented on specific biomarkers related to gastrointestinal (GI) manifestations of ATTR. Fecal calprotectin (FC), a non-invasive biomarker for the detection of intestinal inflammation, has been shown to be elevated in patients with ATTR and GI manifestations, compared to sex and age-matched healthy subjects [140].

CA is also associated with abnormal vascular structure and altered endothelial function [141]. Different studies conducted on patients with both AL-CA and ATTR-CM reported that amyloidogenic proteins generate endothelial dysfunction, suggesting the toxic effects of circulating proteins, by means of induced oxidative stress, before amyloid fibril deposition [142,143]. Reactive oxidative stress determines an increase in the expression of endothelial biomarkers and cell adhesion molecules, such as E-selectin, vascular cell adhesion molecule 1 (VCAM-1), disintegrin, and metalloproteinase with thrombospondin type-1 member 13 (ADAMTS-13) and von Willebrand factor (VWF), which can provide data on the biological mechanism of vascular damage in CA [144,145,146]. Future directions target assessing the potential of VWF, along with prothrombin time, activated partial thromboplastin time, fibrinogen, and D-dimer as biomarkers of pro-thrombosis risk in ATTR amyloidosis.

**Figure 7:**
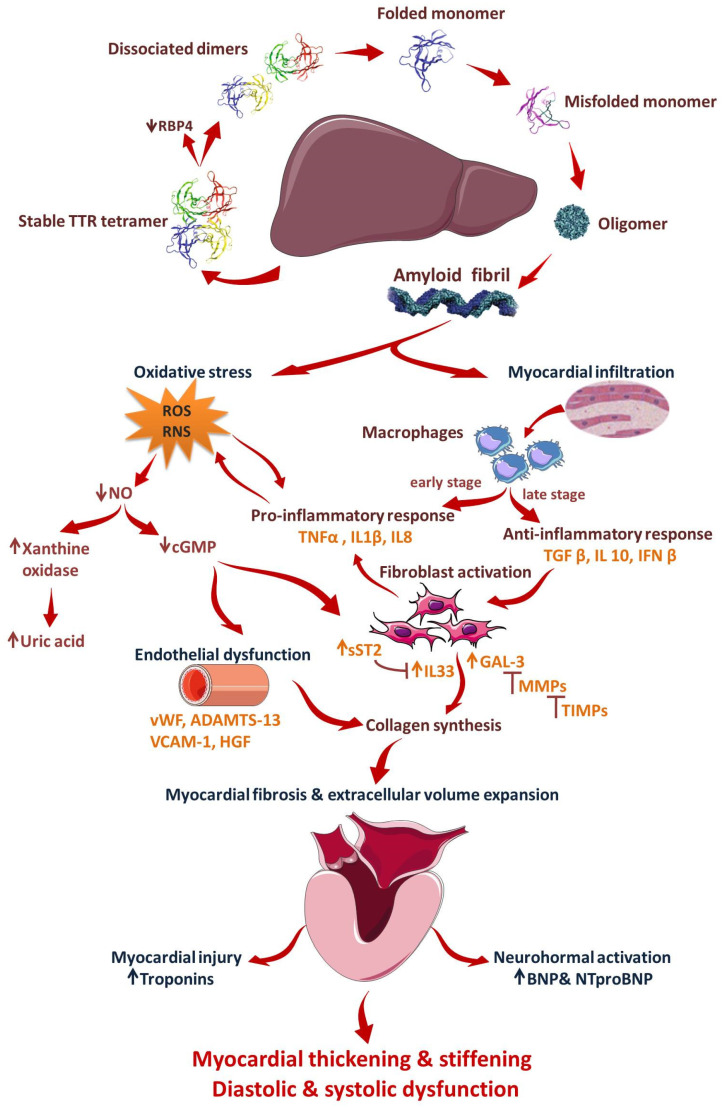
Mechanisms of cardiac impairment in ATTR amyloidosis. Mutations in TTR, a tetrameric protein, lead to its dissociation into dimers, subsequently into monomers that rapidly misfold and aggregate into oligomers, and eventually into amyloid fibrils. Amyloid fibrils infiltrate the myocardial walls, triggering the inflammatory response and fibroblast activation. Amyloid deposits increase oxidative stress, which exacerbates inflammation and induces endothelial dysfunction. These responses lead to myocardial fibrosis and extracellular volume expansion, with myocardial thickening and stiffening and diastolic and systolic dysfunction. TTR: transthyretin; RBP4: retinol binding protein 4; IL: interleukin; TNFα: tumor necrosis factor α; TGFβ:transforming growth factor β; IFN β: interferon β; RO: reactive oxygen species; RNS: reactive nitrogen species; NO: nitric oxide; cGMP: cyclic guanosine monophosphate; sST2: soluble suppression of tumorigenesis 2; Gal-3: galectin-3; MMPs: matrix metallopeptidases; TIMPs: tissue inhibitors of metalloproteinases; vWF: von Willebrand factor; ADAMTS-13: a disintegrin-like and metalloprotease with thrombospondin type 1 repeats-13; VCAM-1: vascular cell adhesion molecule-1; HGF: hepatocyte growth factor (*images for tetrameric proteins and their dissociation products were modified from Coelho T.* et al. *Neurol Ther 5*, *1–25 (2016) with permission under CC BY 4.0)*.

Figure 7 summarizes all potential mechanisms and markers involved in ATTR-CM, from the beginning to the hypertrophic and restrictive remodeling.

## 9. Conclusions

ATTR-CM has an extremely challenging diagnosis and unpredictable outcomes. Compelling evidence supports the role of non-invasive imaging modalities such as 2D STE, CMR, bone tracer CS, SPECT, and PET in the diagnosis of CA. Imaging methods are currently under refinement in order to achieve superior quantification of the disease activity and prediction of prognosis. The diagnosis requires the integration of multimodality cardiac imaging studies, histological, genetic, and hematological assessment, in a step-by-step algorithm. Biomarker-based staging systems for ATTR-CM have not been well validated, thus no single staging system is currently consistently in use. Recent data reinforced the importance of inflammation, oxidative stress, reduced NO availability, thrombosis risk, endothelial dysfunction, and altered vasculature structure, all leading to myocardial remodeling and fibrosis, on top of amyloid deposition. Accordingly, these novel biomarkers might offer incremental diagnostic and prognostic value and identify subjects at high risk of ATTR-CM progression and HF worsening. Extremely limited data are available about imaging and biological parameters to support the utility in longitudinal follow-up.

## Figures and Tables

**Table 1:** Clinical suggestive flags for ATTR modified from Rimbas et al. [37].

Cardiac Amyloidosis	Systemic Involvement
HFpEF with increased LV wall thickness	Carpal tunnel syndrome, particularly if bilateral
HFpEF particularly in older men	Lumbar spinal stenosis (mainly ATTRwt)
Biventricular HF	Spontaneous biceps tendon rupture
HF with intolerance to βblocker, ACEI, or ARB, ARNI	Autonomic dysfunction,
Newly diagnosed HCMP in elderly patients	Orthostatic hypotension,
Paradoxical low flow, low gradient AS in elderly patients	Peripheral neuropathy
Low to normal blood pressure	Deafness
Syncope, conduction, or AV blocks needing pacemaker associated with increased LV wall thickness	Recurrent urinary tract infections
Mild increase in high sensitivity troponin levels (>20 ng/L) on repeated occasions in the absence of coronary artery disease or renal dysfunction	Sexual dysfunction
NT-proBNP, often disproportionately for the degree of HF	Alternating constipation/diarrhea
Low/normal voltage on ECG, with LVH on echocardiography (QRS voltage amplitude <0.5 mV in all limb leads or <1 mV in all precordial leads)	Sweating abnormalities
Pseudo-infarction pattern with no history of myocardial infarction	Unintentional weight loss
Atrioventricular block + LVH + AS (amyloid infiltration of the atrioventricular node)	Pseudo claudication

HF, heart failure; HFpEF, heart failure with preserved ejection fraction; LV, left ventricle; HCMP, hypertrophic cardiomyopathy; ACEI, Angiotensin-Converting Enzyme inhibitors; ARB, Angiotensin II Receptor Blockers; ARNI, Angiotensin Receptor-Neprilysin Inhibitor; AS, aortic stenosis; AV, atrioventricular; NT-proBNP, NT-proB-type Natriuretic Peptide; ECG, electrocardiogram; LVH, left ventricular hypertrophy, QRS, complex on ECG.
